# Correction to: Identifying significantly impacted pathways: a comprehensive review and assessment

**DOI:** 10.1186/s13059-019-1882-1

**Published:** 2019-11-12

**Authors:** Tuan-Minh Nguyen, Adib Shafi, Tin Nguyen, Sorin Draghici

**Affiliations:** 1 Department of Computer Science, Wayne State University, Detroit, 48202 USA; 2 Department of Computer Science and Engineering, University of Nevada, Reno, 89557 USA; 3 Department of Obstetrics and Gynecology, Wayne State University, Detroit, 48202 USA

**Correction to: Genome Biology (2019) 20:203**

**https://doi.org/10.1186/s13059-019-1790-4**

Following publication of the original paper [1], the authors reported the following update to the competing interests declaration:

Competing interest

SD is the founder and CEO of Advaita Bioinformatics, which uses one of the methods benchmarked in this article. SD is also the author of two of the other methods compared in this work.
