# Supplementary material for: Identifying significantly impacted pathways: a comprehensive review and assessment
Source: Genome Biol. 2019 Oct 9;20:203. doi: 10.1186/s13059-019-1790-4 (PMC6784345; doi:10.1186/s13059-019-1790-4)
Supplement: Supplementary file 1 — Supplementary figures and tables. Figure S1. Distribution of numbers of DE genes of 75 human gene expression data sets in the first experiment using the thresholds of corrected p-values <0.05 and log|FC|>1.5. Figure S2. Distribution of numbers of DE genes of 11 mouse gene expression data sets using different thresholds of 1.5 and 5% for log|FC| and corrected p-values, respectively. Figure S3. Comparison of 8 methods using 11 KO data sets in term of accuracy, sensitivity, and specificity. Figure S4. Examples of pathways that have empirical null distributions of p-values biased toward 0. Figure S5. Examples of pathways that have empirical null distributions of p-values biased toward 1. Figure S6. Aggregate p-values of all the pathways generated by GSEA are uniformly distributed under the null. Table S1. 75 benchmark data sets of 15 diseases used to compare 13 methods in this paper. Table S2. Eleven knockout benchmark data sets used to compare 8 methods in this paper. Table S3. Number of methods biased for each pathway. (PDF 6475 kb) [file 13059_2019_1790_MOESM1_ESM.pdf]

Identifying significantly impacted pathways: a comprehensive review and assessment (Supplementary Materials)

Tuan-Minh Nguyen   Adib Shafi   Tin Nguyen   Sorin Drăghici

August 7, 2019

1 Benchmark Data Sets

Table S1 provides detailed information regarding the 75 human data sets used for benchmarking methods' ability to identify target pathways. This information includes: GEO ID, disease, number of normal samples and phenotype samples, Pubmed ID, tissue from which the samples were taken, and the platform used for the experiment.

Table S2 provides detailed information regarding the 11 benchmark KO data sets used. This information includes: the GEO ID, symbol of KO gene, number of truly impacted pathways, number of normal samples, number of phenotype samples, Pubmed ID, tissue from which the samples were taken, and the platform used for the experiment.

All data sets were downloaded from Gene Expression Omnibus database. We normalized them using RMA background adjustment, quantile normalization, and median polish summarization. We used the *threestep* function from *affyPLM* package to perform those steps. Subsequently, standard genome wide annotation packages corresponding to the platform, e.g. hgu133a.db for HG-U133A, were used to map probes to genes. In case there are multiple probes mapped to the same gene, the median value is chosen.

Table S1: 75 benchmark data sets of 15 diseases used to compare 11 methods in this paper.

GEO ID	Disease	#Normal	#Condition	Pubmed ID	Tissue	Platform
GSE781	Renal cell carcinoma	5	12	14641932	Kidney	HG-U133A
GSE14762	Renal cell carcinoma	12	9	19252501	Kidney	HG-U133 Plus 2.0
GSE6357	Renal cell carcinoma	12	6	27063186	CD8+ T Cell	HG-U133A
GSE6344	Renal cell carcinoma	10	10	17699851	Clear cell RCC	HG-U133A
GSE48352	Renal cell carcinoma	8	24	NA	Kidney	HG-U133 Plus 2.0
GSE1297	Alzheimer's disease	9	7	14769913	Hippocampal CA1	HG-U133A
GSE5281EC	Alzheimer's disease	13	10	17077275	Brain, Entorhinal Cortex	HG-U133 Plus 2.0
GSE5281HIP	Alzheimer's disease	13	10	17077275	Brain, hippocampus	HG-U133 Plus 2.0
GSE5281VCX	Alzheimer's disease	12	19	17077275	Brain, primary visual cortex	HG-U133 Plus 2.0
GSE16759	Alzheimer's disease	8	4	20126538	Parietal lobe	HG-U133 Plus 2.0
GSE3467	Thyroid cancer	9	9	16365291	Thyroid	HG-U133 Plus 2.0
GSE3678	Thyroid cancer	7	7	NA	Thyroid	HG-U133 Plus 2.0
GSE58545	Thyroid cancer	18	27	26625260	Thyroid	HG-U133A
GSE85457	Thyroid cancer	3	4	NA	Thyroid	HG-U133 Plus 2.0
GSE58689	Thyroid cancer	18	27	26625260	Thyroid	HG-U133A
GSE3585	Dilated cardiomyopathy	5	7	17045896	Heart, subendocardial left ventricular	HG-U133A
GSE33970	Dilated cardiomyopathy	18	5	NA	Whole blood and heart	HG-U133 Plus 2.0
GSE29819	Dilated cardiomyopathy	12	14	22085907	Heart, left and right ventricular	HG-U133 Plus 2.0
GSE79962	Dilated cardiomyopathy	11	9	NA	Heart	HuGene-10st
GSE21610	Dilated cardiomyopathy	8	42	20460602	Heart	HG-U133 Plus 2.0
GSE4107	Colorectal cancer	10	12	17317818	Colonic mucosa	HG-U133 Plus 2.0
GSE8671	Colorectal cancer	32	32	18171984	Colon	HG-U133 Plus 2.0
GSE9348	Colorectal cancer	12	70	20143136	Colon	HG-U133 Plus 2.0
GSE23878	Colorectal cancer	19	19	21281787	Colon	HG-U133 Plus 2.0
GSE4183	Colorectal cancer	8	15	18776587	Colon	HG-U133 Plus 2.0
GSE6956C	Prostate cancer	11	36	18245496	Prostate	HG-U133A 2
GSE6956AA	Prostate cancer	7	33	18245496	Prostate	HG-U133A 2
GSE55945	Prostate cancer	7	12	19737960	Prostate	HG-U133 Plus 2.0
GSE26910	Prostate cancer	6	6	21611158	Prostate	HG-U133 Plus 2.0
GSE104749	Prostate cancer	4	4	NA	Prostate	HG-U133 Plus 2.0
GSE8762	Huntington's disease	10	12	17724341	Lymphocyte	HG-U133 Plus 2.0
GSE24250	Huntington's disease	6	8	21969577	Venous cellular whole blood	HG-U133A
GSE73655	Huntington's disease	7	13	26756592	Subcutaneous adipose	HuGene-10st
GSE45516	Huntington's disease	3	6	24296361	Fibroblasts	HG-U133 Plus 2.0
GSE37517	Huntington's disease	5	8	22748968	Neural stem cell	HuGene-10st
GSE9476	Acute Myeloid Leukemia	37	26	17910043	Peripheral blood, bone marrow	HG-U133A
GSE14924_CD4	Acute Myeloid Leukemia	10	10	19710498	CD4 T Cell	HG-U133 Plus 2.0
GSE14924_CD8	Acute Myeloid Leukemia	11	10	19710498	CD8 T Cell	HG-U133 Plus 2.0
GSE92778	Acute Myeloid Leukemia	6	6	29035359	Bone marrow stroma cells	HuGene-10st

GSE68172	Acute Myeloid Leukemia	5	72	NA	LSC, HSC and leukemic bulk AML *	HG-U133 Plus 2.0
GSE15471	Pancreatic cancer	35	35	19260470	Pancreas	HG-U133 Plus 2.0
GSE16515	Pancreatic cancer	15	15	19732725	Pancreas	HG-U133 Plus 2.0
GSE32676	Pancreatic cancer	7	25	22261810	Pancreas	HG-U133 Plus 2.0
GSE28735	Pancreatic cancer	45	45	23918603	Pancreas	HuGene-10st
GSE18670	Pancreatic cancer	6	18	23157946	Pancreas	HG-U133 Plus 2.0
GSE18842	Non-small cell lung cancer	44	44	20878980	Lung	HG-U133 Plus 2.0
GSE19188	Non-small cell lung cancer	62	91	20421987	Lung	HG-U133 Plus 2.0
GSE19804	Non-small cell lung cancer	60	60	20802022	Lung	HG-U133 Plus 2.0
GSE50627	Non-small cell lung cancer	6	9	25881239	Lung	HuGene-10st
GSE6044	Non-small cell lung cancer	5	31	18992152	Lung	HG-Focus
GSE19728	Glioma	4	17	21836821	Brain	HG-U133 Plus 2.0
GSE21354	Glioma	4	13	21836821	Brain	HG-U133 Plus 2.0
GSE50161	Glioma	13	95	24078694	Brain	HG-U133 Plus 2.0
GSE4290	Glioma	23	157	16616334	Brain	HG-U133 Plus 2.0
GSE44971	Glioma	9	49	23660940	Brain	HG-U133 Plus 2.0
GSE20153	Parkinson's disease	8	8	20926834	B lymphocytes from peripheral blood	HG-U133 Plus 2.0
GSE20291	Parkinson's disease	20	15	15965975	Brain	HG-U133A
GSE20164	Parkinson's disease	5	6	20926834	Substantia nigra (midbrain)	HG-U133A
GSE7621	Parkinson's disease	9	16	17571925	Substantia nigra (midbrain)	HG-U133 Plus 2.0
GSE19587	Parkinson's disease	10	12	20837543	Brain	HG-U133A 2
GSE19420	Type II diabetes mellitus	12	12	22802091	Skeletal muscle vastus lateralis	HG-U133 Plus 2.0
GSE39825	Type II diabetes mellitus	6	4	23919306	Fibroblasts (cell culture)	HG_U95Av2
GSE26887	Type II diabetes mellitus	5	7	22427379	Left ventricle	HuGene-10st
GSE21340	Type II diabetes mellitus	15	5	23919306	Skeletal muscle	HG_U95Av2
GSE38642	Type II diabetes mellitus	54	9	22768844	Pancreatic islets	HuGene-10st
GSE24739_G0	Chronic Myeloid Leukemia	4	8	21436996	Peripheral blood	HG-U133 Plus 2.0
GSE24739_G1	Chronic Myeloid Leukemia	4	8	21436996	Peripheral blood	HG-U133 Plus 2.0
GSE33075	Chronic Myeloid Leukemia	18	9	22388797	Bone marrow	HG-U133 Plus 2.0
GSE24739	Chronic Myeloid Leukemia	8	16	21436996	Peripheral blood and bone marrow	HG-U133 Plus 2.0
GSE1418	Chronic Myeloid Leukemia	6	8	15618956	Bone marrow	HG-Focus
GSE7305	Endometrial cancer	10	10	17640886	Endometrium/Ovarian tissue	HG-U133 Plus 2.0
GSE63678	Endometrial cancer	5	7	26559525	Endometrium	HG-U133A
GSE7803	Endometrial cancer	10	31	17974957	Cervix and squamous cervical epitheilium	HG-U133A
GSE17025	Endometrial cancer	12	91	21619611	Endometrium	HG-U133 Plus 2.0
GSE36389	Endometrial cancer	7	13	NA	Endometrium	HG-U133A

*Leukemic stem cells (LSC), hematopoietic stem cells (HSCs), and AML bulk cells (CD34+CD38+, CD34-CD38+ and CD34-CD38)

Table S2: 11 knock-out benchmark data sets used to compare 8 methods in this paper.

**GEO ID**	**KO gene**	**#Impacted Pathways**	**#Nornal**	**#Condition**	**Pubmed ID**	**Tissue**	**Platform**
GSE22873	Myd88	19	11	8	22075646	Liver	Mouse430_2
GSE6030	Neurod1	1	3	3	17630985	Pineal gland	Mouse430_2
GSE29048	Pdx1	3	4	4	22135308	Intestinal epithelium	Mouse430_2
GSE70302	IL1a	20	4	4	26224856	Spinal cord	MoGene-1_0-st
GSE70302	IL1b	34	4	4	26224856	Spinal cord	MoGene-1_0-st
GSE58120	IL2	3	6	6	25652593	Myeloid dendritic cells	MoGene-1_0-st
GSE46211	TGFBR2	20	12	6	24496627	Anterior palatal tissue & posterior palatal tissue	Mouse430_2
GSE49166	BHLHE40	1	3	3	24699451	CD4 T cells	MoGene-1_0-st
GSE50933	ID3	2	5	5	24244015	Natural killer T cells	Mouse430_2
GSE62999	DUSP5	1	10	10	25398911	Bone marrow	Mouse430_2
GSE57917	ONECUR1	2	3	3	25313862	Retinas	Mouse430_2

2 Problems with Classical Selection of DE Genes

Setting thresholds based on their p-values and unsigned log-fold changes is a widely used method to obtain a list of DE genes. However, the numbers of DE genes obtained from different studies of the same condition often differ significantly due to the heterogeneity present in the individual experiments. For example, with the thresholds of 1.5 for unsigned log-fold changes and and 5% for the corrected p-values, 21 out of 75 human gene expression data sets studied do not have any DE gene, whereas one data set has more than one thousand DE genes (Fig. S1). A similar problem occurs with the 11 KO data sets, 5 of which do not have any DE gene according to these criteria (Fig. S2).

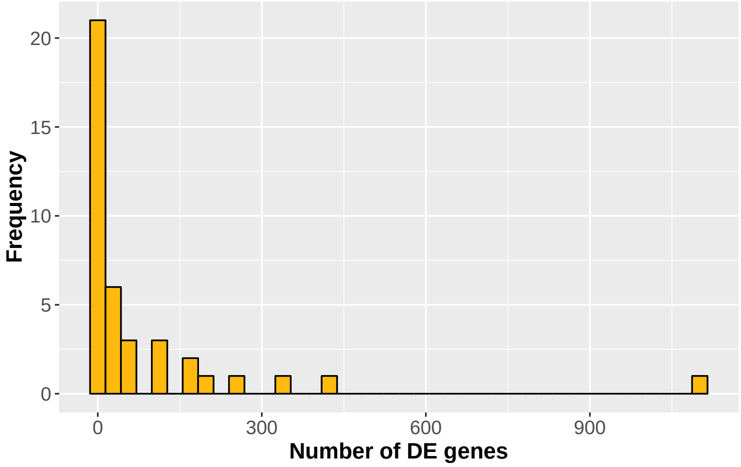

Fig. S1: **Distribution of number of DE genes of 75 human gene expression data sets using corrected p-value threshold of 0.05 and unsigned log-fold change threshold of 1.5.** The number of DE genes varies considerably across all the data sets. In fact, 21 data sets do not have any DE genes whereas there is one data set that has more than 1000 DE genes.

Here, to eliminate the effect of the thresholds, we select the same number of DE genes for each experiment. This is consistent with the findings of the MAQC consortium which reported that the best reproducibility across labs and platforms is obtained when genes are selected based on their fold changes [1, 2]. The procedure to select the DE genes was as follows. First, we calculated the gene level p-values using the two sample t-test. Subsequently, we selected genes with p-values less than 5%. Finally, the top 400 (around 10% number of genes present in KEGG) genes with the highest unsigned log-fold changes were considered as DE genes.

3 Accuracy, sensitivity, and specificity

KO data sets are used to calculate the statistical measures of 10 methods (CePaGSA, CePaORA, and PathNet are not included in this comparison because they do not support mouse pathways). After defining the true positives, true negatives, false positives, and false negatives, the accuracy, sensitivity, specificity, and the AUC are measured using formula in sub-section "Statistical measures". In this supplementary we plotted only the former three measures into Fig. S3. ROntoTools and PADOG have the highest median value of accuracy (0.91). ROntoTools also has the highest median value of specificity (0.94). All of the methods show rather low sensitivity. Among them, KS is the best one with the median value of sensitivity of 0.2.

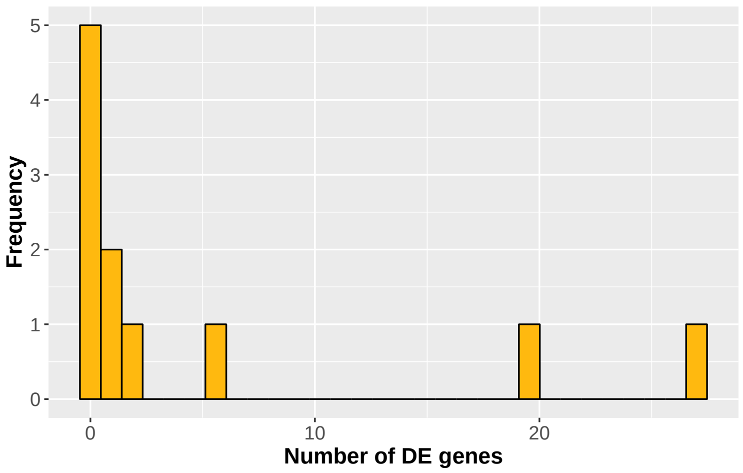

Fig. S2: **Distribution of number of DE genes of 11 mouse gene expression data sets using corrected p-value threshold of 0.05 and unsigned log-fold change threshold of 1.5** Five of them do not have any DE genes.

4 Empirical null distributions

Pathway analysis methods work under an assumption that empirical null distributions of p-values of all pathways are uniformly distributed under the true null hypothesis. However, this does not hold true in most of the cases. Fig. S4 and Fig. S5 show some examples of pathways that have empirical null distribution of p-values as reported by various methods, biased toward 0 and 1, respectively.

GSEA is the only method in this study that is unbiased for all the pathways. Fig. S6 shows that the aggregate p-values of all pathways generated by GSEA are uniformly distributed.

5 Number of methods biased for each pathway

While benchmarking pathway analysis methods, it is important to choose appropriate data sets. In a fair comparison, the target pathways related to the disease or condition of these data sets should have unbiased null distributions of p-value produced by all methods studied. If the null-distribution of p-values of a target pathway is not available, knowing the probability of that pathway being biased toward 0 or 1 is also helpful. In an attempt to provide this information, for each pathway we report the number of methods (out of the 11 methods investigated) biased toward 0 or 1 (Table S3).

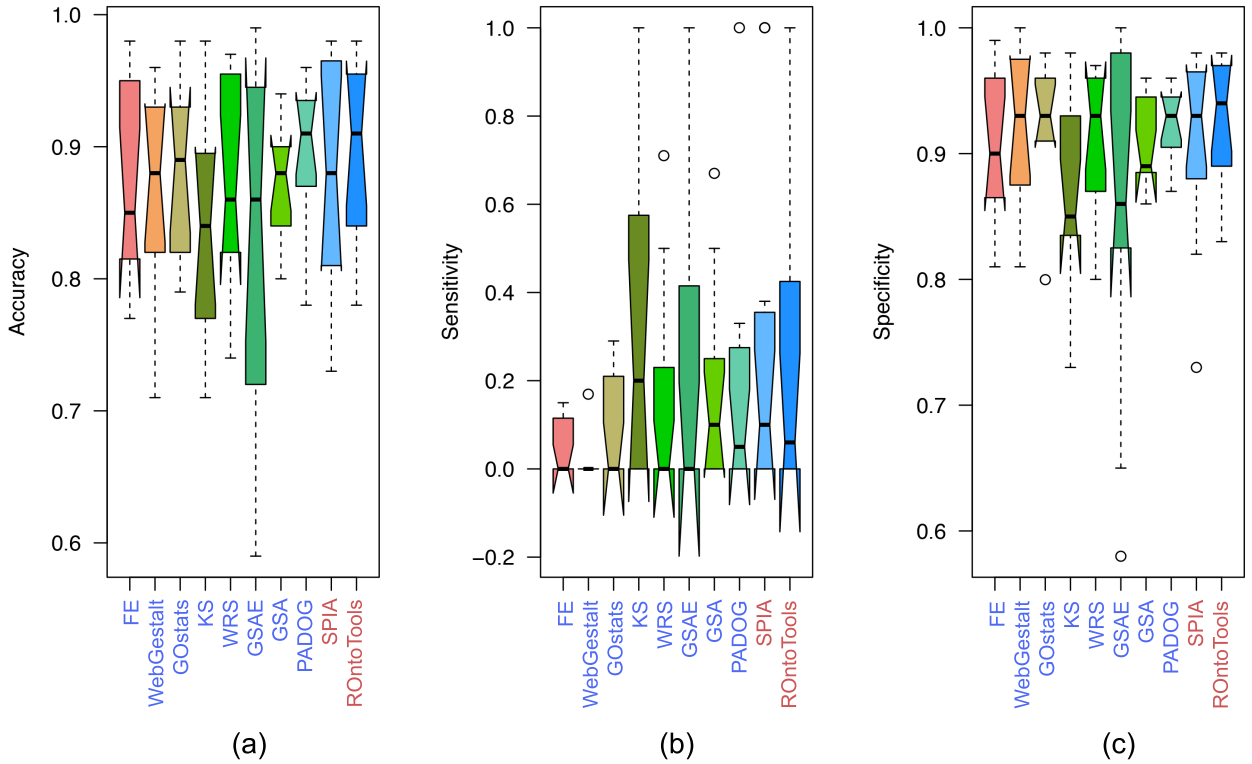

Fig. S3: **Comparison of 8 methods using 11 KO data sets in term of accuracy (a), sensitivity (b), and specificity (c).** In term of accuracy, ROntoTools and PADOG have the highest median value (0.91). ROntoTools also has the highest median value of specificity (0.94). The best method in term of sensitivity is KS which has the median value of sensitivity of 0.2. However, KS also has the lowest median specificity.

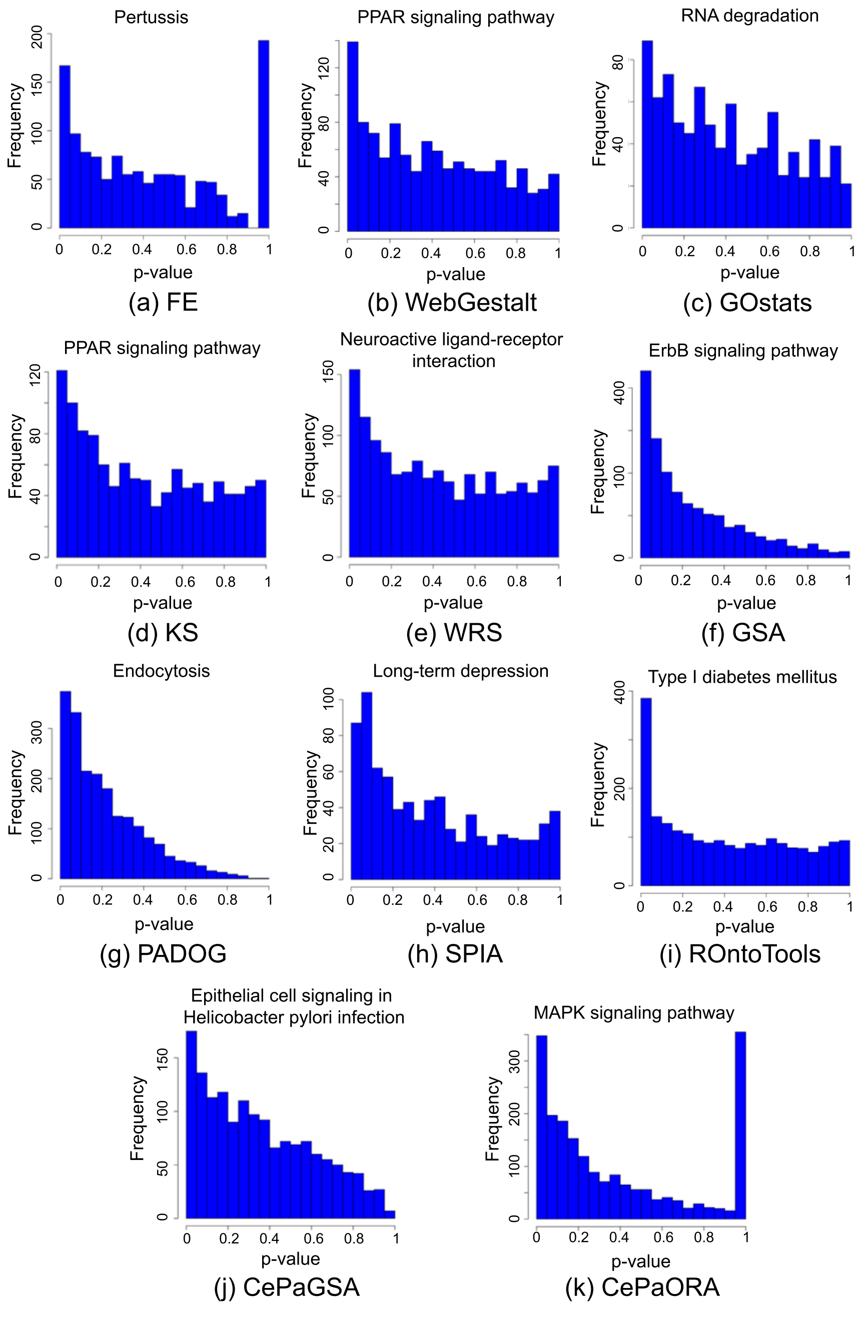

Fig. S4: **Examples of pathways that have empirical null distributions of p-value biased toward 0.** The procedure for generating null distributions is described in Fig. 5. The x-axes display the p-values whereas the y-axes display the frequencies. These pathways are likely to be falsely identified as significantly impacted by the corresponding method (false positive).

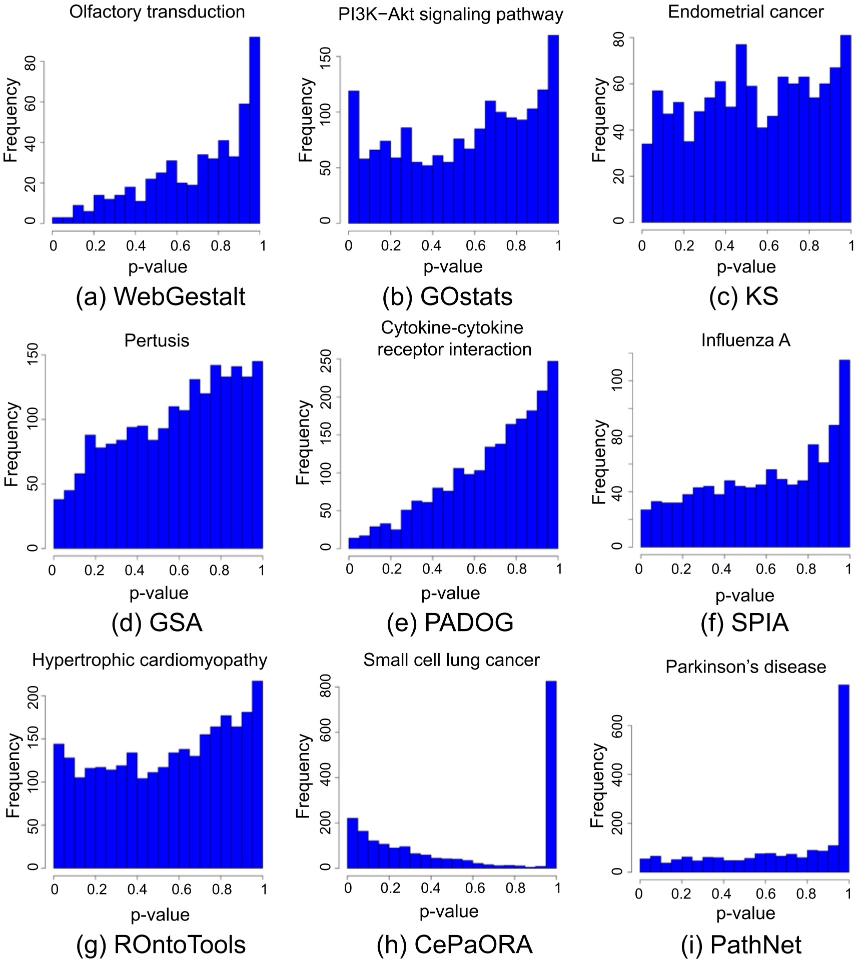

Fig. S5: **Examples of pathways that have empirical null distributions of p-value biased toward 1.** In these sub-figures, x-axes represent the p-value, while y-axes represent their frequencies. These pathways are often incorrectly excluded in the list of significant pathways by the corresponding method even when they are indeed impacted (false negative).

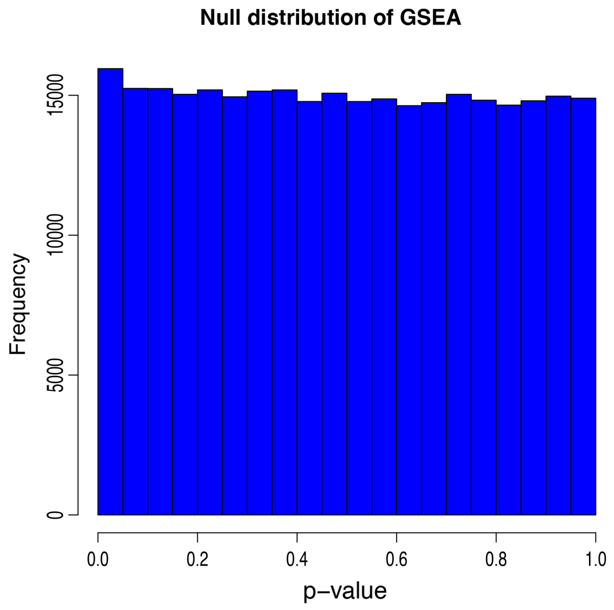

Fig. S6: **Aggregate p-values of all the pathways generated by GSEA are uniformly distributed under the null.** The uniform distribution proves that GSEA is extremely unbiased.

Table S3: Number of methods biased for each pathway

Pathway ID	Pathway Names	Bias toward 0	Bias toward 1	Total
hsa04390	Hippo signaling pathway	3	0	3
hsa04066	HIF-1 signaling pathway	3	0	3
hsa04530	Tight junction	3	1	4
hsa05166	HTLV-I infection	5	0	5
hsa04670	Leukocyte transendothelial migration	5	0	5
hsa05142	Chagas disease (American trypanosomiasis)	4	1	5
hsa04514	Cell adhesion molecules (CAMs)	4	1	5
hsa04310	Wnt signaling pathway	4	1	5
hsa04151	PI3K-Akt signaling pathway	4	1	5
hsa05034	Alcoholism	2	3	5
hsa05169	Epstein-Barr virus infection	6	0	6
hsa05215	Prostate cancer	5	1	6
hsa05212	Pancreatic cancer	5	1	6
hsa05202	Transcriptional misregulation in cancer	5	1	6
hsa05161	Hepatitis B	5	1	6
hsa05030	Cocaine addiction	5	1	6
hsa04810	Regulation of actin cytoskeleton	5	1	6
hsa04726	Serotonergic synapse	5	1	6
hsa04713	Circadian entrainment	5	1	6
hsa04540	Gap junction	5	1	6
hsa04370	VEGF signaling pathway	5	1	6
hsa04270	Vascular smooth muscle contraction	5	1	6
hsa04064	NF-kappa B signaling pathway	5	1	6
hsa05203	Viral carcinogenesis	4	2	6
hsa05164	Influenza A	4	2	6
hsa05162	Measles 4	2	6	
hsa05152	Tuberculosis	4	2	6
hsa05120	Epithelial cell signaling in Helicobacter pylori infection	4	2	6
hsa04916	Melanogenesis	4	2	6
hsa04727	GABAergic synapse	4	2	6
hsa04723	Retrograde endocannabinoid signaling	4	2	6
hsa04330	Notch signaling pathway	4	2	6
hsa04210	Apoptosis	4	2	6
hsa03460	Fanconi anemia pathway	4	2	6
hsa04920	Adipocytokine signaling pathway	3	3	6
hsa04144	Endocytosis	3	3	6
hsa04914	Progesterone-mediated oocyte maturation	7	0	7
hsa05214	Glioma	6	1	7
hsa05168	Herpes simplex infection	6	1	7
hsa04725	Cholinergic synapse	6	1	7
hsa04724	Glutamatergic synapse	6	1	7
hsa04721	Synaptic vesicle cycle	6	1	7
hsa04664	Fc epsilon RI signaling pathway	6	1	7
hsa04380	Osteoclast differentiation	6	1	7
hsa04360	Axon guidance	6	1	7
hsa05323	Rheumatoid arthritis	5	2	7
hsa05218	Melanoma	5	2	7
hsa05210	Colorectal cancer	5	2	7
hsa05132	Salmonella infection	5	2	7
hsa04340	Hedgehog signaling pathway	5	2	7
hsa04010	MAPK signaling pathway	5	2	7
hsa03008	Ribosome biogenesis in eukaryotes	5	2	7
hsa05032	Morphine addiction	4	3	7
hsa04620	Toll-like receptor signaling pathway	4	3	7
hsa05016	Huntington's disease	3	4	7
hsa04650	Natural killer cell mediated cytotoxicity	3	4	7

hsa04961	Endocrine and other factor-regulated calcium reabsorption	8	0	8
hsa05222	Small cell lung cancer	7	1	8
hsa05145	Toxoplasmosis	7	1	8
hsa05031	Amphetamine addiction	7	1	8
hsa04912	GnRH signaling pathway	7	1	8
hsa04666	Fc gamma R-mediated phagocytosis	7	1	8
hsa04662	B cell receptor signaling pathway	7	1	8
hsa04350	TGF-beta signaling pathway	7	1	8
hsa05200	Pathways in cancer	6	2	8
hsa05160	Hepatitis C	6	2	8
hsa04520	Adherens junction	6	2	8
hsa05217	Basal cell carcinoma	5	3	8
hsa05134	Legionellosis	5	3	8
hsa05133	Pertussis	5	3	8
hsa05010	Alzheimer's disease	5	3	8
hsa04973	Carbohydrate digestion and absorption	5	3	8
hsa04145	Phagosome	5	3	8
hsa04020	Calcium signaling pathway	5	3	8
hsa05322	Systemic lupus erythematosus	4	4	8
hsa04622	RIG-I-like receptor signaling pathway	4	4	8
hsa04142	Lysosome	4	4	8
hsa05100	Bacterial invasion of epithelial cells	8	1	9
hsa04710	Circadian rhythm	8	1	9
hsa04130	SNARE interactions in vesicular transport	8	1	9
hsa04012	ErbB signaling pathway	8	1	9
hsa03015	mRNA surveillance pathway	8	1	9
hsa05412	Arrhythmogenic right ventricular cardiomyopathy (ARVC)	7	2	9
hsa05223	Non-small cell lung cancer	7	2	9
hsa05220	Chronic myeloid leukemia	7	2	9
hsa05211	Renal cell carcinoma	7	2	9
hsa05130	Pathogenic Escherichia coli infection	7	2	9
hsa04971	Gastric acid secretion	7	2	9
hsa04960	Aldosterone-regulated sodium reabsorption	7	2	9
hsa04910	Insulin signaling pathway	7	2	9
hsa04730	Long-term depression	7	2	9
hsa04728	Dopaminergic synapse	7	2	9
hsa04720	Long-term potentiation	7	2	9
hsa04150	mTOR signaling pathway	7	2	9
hsa04114	Oocyte meiosis	7	2	9
hsa04110	Cell cycle	7	2	9
hsa05416	Viral myocarditis	6	3	9
hsa05332	Graft-versus-host disease	6	3	9
hsa05221	Acute myeloid leukemia	6	3	9
hsa05219	Bladder cancer	6	3	9
hsa05216	Thyroid cancer	6	3	9
hsa05146	Amoebiasis	6	3	9
hsa05143	African trypanosomiasis	6	3	9
hsa05014	Amyotrophic lateral sclerosis (ALS)	6	3	9
hsa04978	Mineral absorption	6	3	9
hsa04940	Type I diabetes mellitus	6	3	9
hsa04621	NOD-like receptor signaling pathway	6	3	9
hsa05410	Hypertrophic cardiomyopathy (HCM)	5	4	9
hsa05140	Leishmaniasis	5	4	9
hsa05012	Parkinson's disease	5	4	9
hsa04970	Salivary secretion	5	4	9
hsa04742	Taste transduction	5	4	9
hsa04630	Jak-STAT signaling pathway	4	5	9
hsa05131	Shigellosis	8	2	10
hsa04722	Neurotrophin signaling pathway	8	2	10

hsa04660	T cell receptor signaling pathway	8	2	10
hsa04512	ECM-receptor interaction	8	2	10
hsa04115	p53 signaling pathway	8	2	10
hsa05213	Endometrial cancer	7	3	10
hsa04950	Maturity onset diabetes of the young	7	3	10
hsa04122	Sulfur relay system	7	3	10
hsa05310	Asthma	6	4	10
hsa04976	Bile secretion	6	4	10
hsa04972	Pancreatic secretion	6	4	10
hsa04612	Antigen processing and presentation	6	4	10
hsa04062	Chemokine signaling pathway	6	4	10
hsa04060	Cytokine-cytokine receptor interaction	6	4	10
hsa05414	Dilated cardiomyopathy	5	5	10
hsa04740	Olfactory transduction	5	5	10
hsa04140	Regulation of autophagy	5	5	10
hsa04962	Vasopressin-regulated water reabsorption	9	2	11
hsa04930	Type II diabetes mellitus	9	2	11
hsa04510	Focal adhesion	9	2	11
hsa05150	Staphylococcus aureus infection	8	3	11
hsa04320	Dorso-ventral axis formation	8	3	11
hsa04141	Protein processing in endoplasmic reticulum	8	3	11
hsa03018	RNA degradation	8	3	11
hsa03013	RNA transport	8	3	11
hsa05330	Allograft rejection	7	4	11
hsa05110	Vibrio cholerae infection	7	4	11
hsa04744	Phototransduction	7	4	11
hsa04672	Intestinal immune network for IgA production	7	4	11
hsa04610	Complement and coagulation cascades	7	4	11
hsa04260	Cardiac muscle contraction	7	4	11
hsa05020	Prion diseases	6	5	11
hsa03320	PPAR signaling pathway	6	5	11
hsa04080	Neuroactive ligand-receptor interaction	4	7	11
hsa05320	Autoimmune thyroid disease	7	5	12
hsa05144	Malaria	7	5	12
hsa04623	Cytosolic DNA-sensing pathway	7	5	12

References

[1] MAQC Consortium: The MicroArray Quality Control (MAQC) project shows inter- and intraplatform reproducibility of gene expression measurements. Nature Biotechnology **24**(9), 1151–1161 (2006)

[2] Chen, J.J., Hsueh, H.-M., Delongchamp, R.R., Lin, C.-J., Tsai, C.-A.: Reproducibility of microarray data: a further analysis of microarray quality control (MAQC) data. BMC Bioinformatics **8**(1), 412 (2007)
